# Neoadjuvant chemoradiation with Gemcitabine for locally advanced pancreatic cancer

**DOI:** 10.1186/1748-717X-7-28

**Published:** 2012-03-02

**Authors:** Daniel Habermehl, Kerstin Kessel, Thomas Welzel, Holger Hof, Amir Abdollahi, Frank Bergmann, Stefan Rieken, Jürgen Weitz, Jens Werner, Peter Schirmacher, Markus W Büchler, Jürgen Debus, Stephanie E Combs

**Affiliations:** 1 Department of Radiation Oncology, Im Neuenheimer Feld 400, 69120 Heidelberg, Germany; 2 Department of Visceral Surgery, Im Neuenheimer Feld 110, 69120 Heidelberg, Germany; 3 Institute of Pathology, Im Neuenheimer Feld 220/221, 69120 Heidelberg, Germany; 4 Department of Radiation Oncology, University Hospital of Heidelberg, Im Neuenheimer Feld 400, 69120 Heidelberg, Germany

## Abstract

**Introduction:**

To evaluate efficacy and secondary resectability in patients with locally advanced pancreatic cancer (LAPC) treated with neoadjuvant chemoradiotherapy (CRT).

**Patients and methods:**

A total of 215 patients with locally advanced pancreatic cancer were treated with chemoradiation at a single institution. Radiotherapy was delivered with a median dose of 52.2 Gy in single fractions of 1.8 Gy. Chemotherapy was applied concomitantly as gemcitabine (GEM) at a dose of 300 mg/m^2 ^weekly, followed by adjuvant cycles of full-dose GEM (1000 mg/m^2^). After neoadjuvant CRT restaging was done to evaluate secondary resectability. Overall and disease-free survival were calculated and prognostic factors were estimated.

**Results:**

After CRT a total of 26% of all patients with primary unresectable LAPC were chosen to undergo secondary resection. Tumour free resection margins could be achieved in 39.2% (R0-resection), R1-resections were seen in 41.2%, residual macroscopic tumour in 11.8% (R2) and in 7.8% resection were classified as Rx. Patients with complete resection after CRT showed a significantly increased median overall survival (OS) with 22.1 compared to 11.9 months in non-resected patients. Median OS and disease-free survival (DFS) of all patients were 12.3 and 8.1 months respectively. In most cases the first site of disease progression was systemic with hepatic (52%) and peritoneal (36%) metastases.

**Discussion:**

A high percentage of patients with locally advanced pancreatic cancer can undergo secondary resection after gemcitabine-based chemoradiation and has a relative long-term prognosis after complete resection.

## Introduction

To date, the minority of patients with pancreatic tumours is amenable to curative resection. Facing an increasing incidence in pancreatic cancer, optimization of treatment with extended disease-free and overall survival (DFS and OS) while preserving quality of life is a special focus in patients with locally advanced pancreatic cancer (LAPC).

In 80-90% of all patients diagnosed with pancreatic cancer, locally advanced disease associated with regional lymph node invasion or systematically disseminated disease can be observed [1-5]. Therefore, only about 10-20% of all tumours can be primarily resected, and even in resected patients only about 20% of all patients survive for more than 5 years [6-10]. Since the strongest prognostic factor for outcome is the extent of resection, and tumour-free (R0) resection is the ultimate goal, strategies to increase resectability and to enable the surgeon to achieve complete resections are of special interest [11,12]. Neoadjuvant radiation combined with chemotherapy has been proven to be effective and to lead to substantial downstaging prior to surgery, associated with improved outcome, for several tumour entities [4,5,13-17]. For pancreatic cancer, similar approaches have been introduced, with controversial clinical data. Two recent meta-analyses have shown substantial benefit of preoperative chemoradiation in locally advanced pancreatic cancer [18,19]. For CRT there is no consensus on whether to use Gemcitabine (GEM) or 5-fluorouracil (5-FU) in pancreatic carcinoma. GEM is a nucleoside analogue which exerts its anti-tumour activity by incorporation of a metabolite into DNA-strands during replication and thus leading to a growth inhibition of the cell [20]. Furthermore GEM is a potent radiosensitizer with impact on cell cycle effects and cell death pathways [21].

The present study, evaluates outcome and rate of secondary resectability in a large group of patients treated with a homogeneous radiation and chemotherapy protocol.

## Materials and methods

### Patients characteristics

From 2001 to 2010 a total of 215 consecutive patients with locally advanced and inoperable pancreatic cancer were treated with chemoradiotherapy (CRT) including radiotherapy and concurrent chemotherapy with GEM. All patients were evaluated in an interdisciplinary setting including all relevant specialties.

Seventeen patients were excluded from analysis because of the following reasons: recurrent pancreatic carcinoma, newly detected distant metastasis during initiation of CRT, treatment only with intraoperative radiotherapy in our institution (combined CRT in another facility), non-suitability for surgery because of age (n = 1) or liver cirrhosis (n = 1). The median age was 67 years (range 42-93 years) and 110 patients were male and 88 female. Patient characteristics are shown in Table 1.

**Table 1:** Patient and treatment details

	Number (%)	
	**(n = 215)**	

Excluded from analysis	17	

**Gender**		

Male	110	(56%)

Female	88	(44%)

**Age (median, range)**	67	(42 - 93)

**Tumour Location**		

(tumours may involve more than 1 region)		

Head	114	(58%)

Head and Body	27	(14%)

Body	36	(18%)

Body and Tail	3	(2%)

Tail	6	(3%)

Head, Body and Tail	5	(3%)

No information	7	(4%)

**Radiotherapy**		

< 50 Gy	30	(16%)

≥ 50 Gy	160	(84%)

Median Dose (range)	52.2 Gy	(39.6 - 54.4 Gy)

**Previous Chemotherapy**		

All	23	(12%)

Gemcitabine mono	11	(6%)

Gemcitabine-containing regimen	8	(5%)

**Concomitant Chemotherapy**		

All	198	

Gemcitabine-containing regimen	198	(100%)

Gemcitabine mono	194	(97.5%)

Gemcitabine + 5-FU or Capecitabine	3	(1.5%)

Gemcitabine + Cisplatin	1	(0.5%)

**IORT After Tumor Resection**	26	(51%) (n = 51)

**IORT - Doses**	(n = 26)	

10 Gy	1	(4%)

12 Gy	4	(15%)

15 Gy	21	(81%)

*IORT*, intra-operative radiotherapy

Combined CRT was indicated in cases of locally advanced non-metastasized pancreatic carcinoma. Before therapy was initiated a biopsy of the tumor was performed with the histology of ductal adenocarcinoma. If a biopsy was not possible or the acquired specimen was too small for pathological assessment, elevated levels of CA-19-9 and a CT scan clearly showing a pancreatic mass were accepted as a proof of pancreatic cancer. In some patients without histological confirmation material obtained during subsequent resection after CRT confirmed the diagnosis of pancreatic cancer. Criteria for non-resectability were a more than 180 degrees encasement of the superior mesenteric artery, infiltration of the celiac trunk, an unreconstructible occlusion of the superior mesenteric vein or portal vein, aortic invasion or a surrounding tumour of parts of the abdominal aorta.

### Radiation and chemotherapy

For treatment planning, CT imaging was performed according to an in-house standard protocol in supine position. 3-D treatment planning was performed in all patients. GEM i.v. was applied weekly during radiation at a dose of 300 mg/m^2^. After completion of chemoradiation, adjuvant cycles of GEM were applied with full dose levels of 1000 mg/m^2 ^until resectability or disease progression. Two patients received 5-fluorouracil additionally and 1 patient received capecitabine during CRT together with GEM. Before application of GEM, blood values were examined; leucocytes were required to exceed 3.000/μl and platelets to exceed 100.000/μl. Furthermore, physical examination was performed to exclude any evidence of severe infection. During combined CRT and during the adjuvant cycles blood cell count and a physical examination was conducted weekly.

Intraoperative radiotherapy was applied in 26 patients with a median dose of 15 Gy (range 10-15 Gy) (Table 1).

### Assessment of response

Two to six weeks after completion of chemoradiation and after 1 cycle of full-dose GEM evaluation of treatment response and assessment of resectability was performed. In most patients contrast-enhanced computed tomography (CT) or magnetic resonance imaging (MRI) was conducted. Seven patients underwent surgery without previous diagnostic restaging. Decision on surgical resection was made in the interdisciplinary setting.

In patients not classified as candidates for secondary surgical resection, but presenting with stable disease, GEM full dose was continued, and re-assessment of the tumour status was performed continuously.

### Surgery and pathological workup

After response to CRT on radiographic imaging or in cases where response was suspected to have occurred without evident confirmation on imaging, surgery or explorative interventions aiming at resection were performed in our institution. Resected specimen were prepared and diagnosed as primarily described [22].

### Follow-up and statistics

All patients were followed on a regular basis after CRT and response was classified according to the RECIST criteria (Response Evaluation Criteria In Solid Tumours, version 1.1, 2009). Besides the application of adjuvant cycles of full-dose GEM, regular imaging examinations included contrast-enhanced CT- or MR-imaging of the abdomen and analysis of tumour markers.

Overall survival was calculated from the first day of irradiation until death. Disease-free survival (DFS) was calculated from the first day of radiotherapy until documented progression of disease (local or distant metastasis). The log-rank test was implemented to compare survival curves evaluating the association between clinical variables of interest and survival. All calculations were performed using the statistical software program SPSS 18.0 for Windows (Chicago, Illinois, US).

The study is in compliance with the Helsinki Declaration (Sixth Revision, 2008). Furthermore our study was approved by the Institutional Review Board/the independent Ethics Committee of the Medical Faculty Heidelberg (ref. nr.: S-483/2011).

## Results

### Response and resectability after chemoradiation

**Figure 1:**
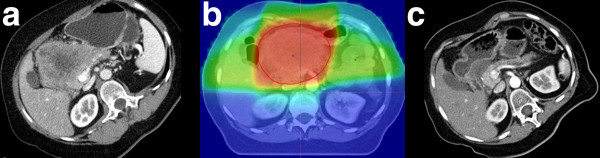
**CT scan of a female patient before (A) and after (C) neoadjuvant CRT showing a partial remission of the pancreatic tumour**. Dose distribution of the applied treatment plan (B).

Response assessment after CRT and adjuvant cycles of full-dose GEM was performed to re-evaluate resectability. According to RECIST criteria, at first follow-up, 11% of the patients had progressive disease (PD). In 9% a partial remission (PR) was observed, and 80% of the patients presented with stable disease (SD). Seven patients did not receive another CT or MRI scan before surgery and were considered for resection or at least explorative laparotomy directly after CRT. Figure 1 depicts a partial remission of a large inoperable tumour due to CRT with GEM.

Secondary espectability was decided in 51 cases (26%), and in an additional 53 patients (28%) surgery was scheduled but was performed as explorative laparotomy (28%) because of intraoperative non-resectability of the primary tumour (n = 30 (58%)) or new diagnosis of metastasis (n = 23 (42%)). In one patient complete tumour resection with negative margins could be achieved in case of tumour-associated duodenal bleeding but new peritoneal and liver metastases were detected (classified as R2-resection).

Among the patients amenable to secondary resection, tumour-free resection margins were achieved in 39,2% (R0), microscopically positive resection margins were diagnosed in 41,2% (R1), incomplete resection with macroscopically positive resection margins in 11,8% (R2) and pathologically not definable margins were seen in 7,8% (Rx).

### Influence of resection status on overall survival (OS)

**Figure 2:**
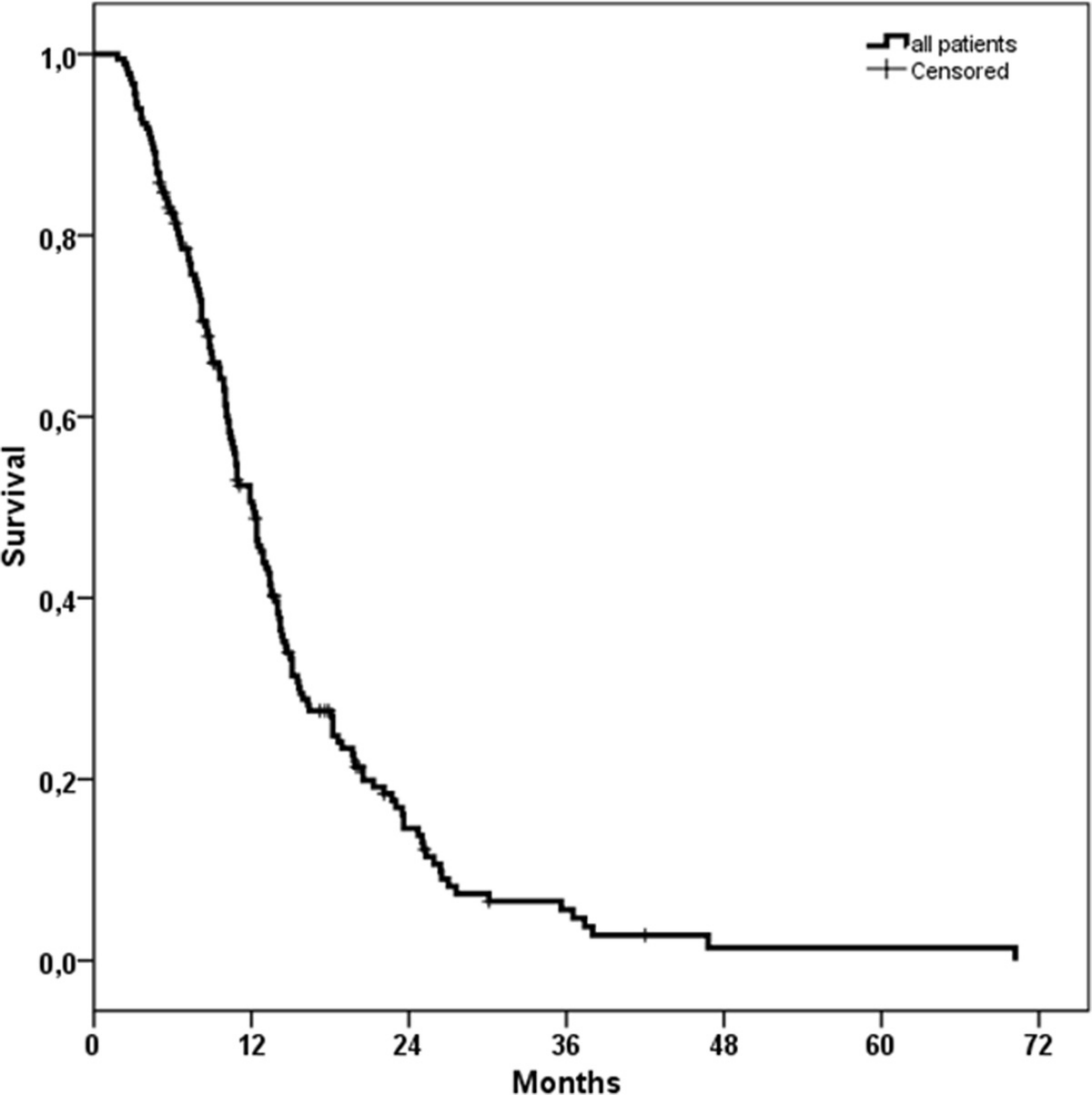
**Kaplan-Meier estimate of overall survival (OS) of all patients**.

Median OS of all patients was 12.3 months (95%-CI: 10.8-13.7 months, SD = 0.73). OS was 82% at 6 months, 46% at 1 and 9% at 2 years (Figure 2).

**Figure 3:**
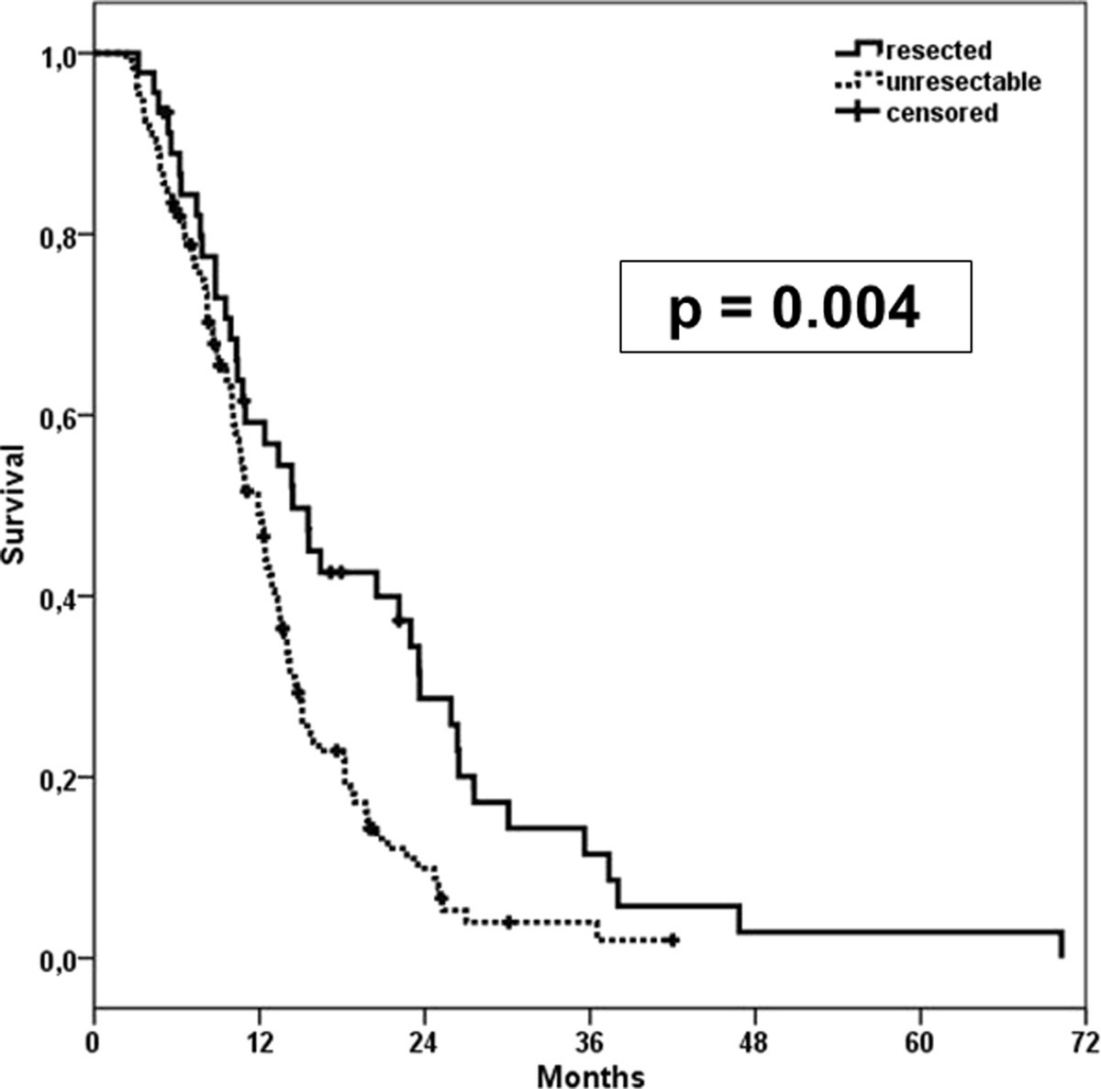
**Kaplan-Meier estimates comparing survival of resected patients and non-resected patients after neoadjuvant CRT**.

Secondary resectability was associated with better OS (p = 0.004; Figure 3). Median OS in non-resected patients was 11.9 months (95%-CI: 10.5-13.3 months, SD = 0.69), and after secondary resection was at a median of 14.4 months (95%-CI: 10.4-18.4 months, SD = 2.1) (Figure 3).

**Figure 4:**
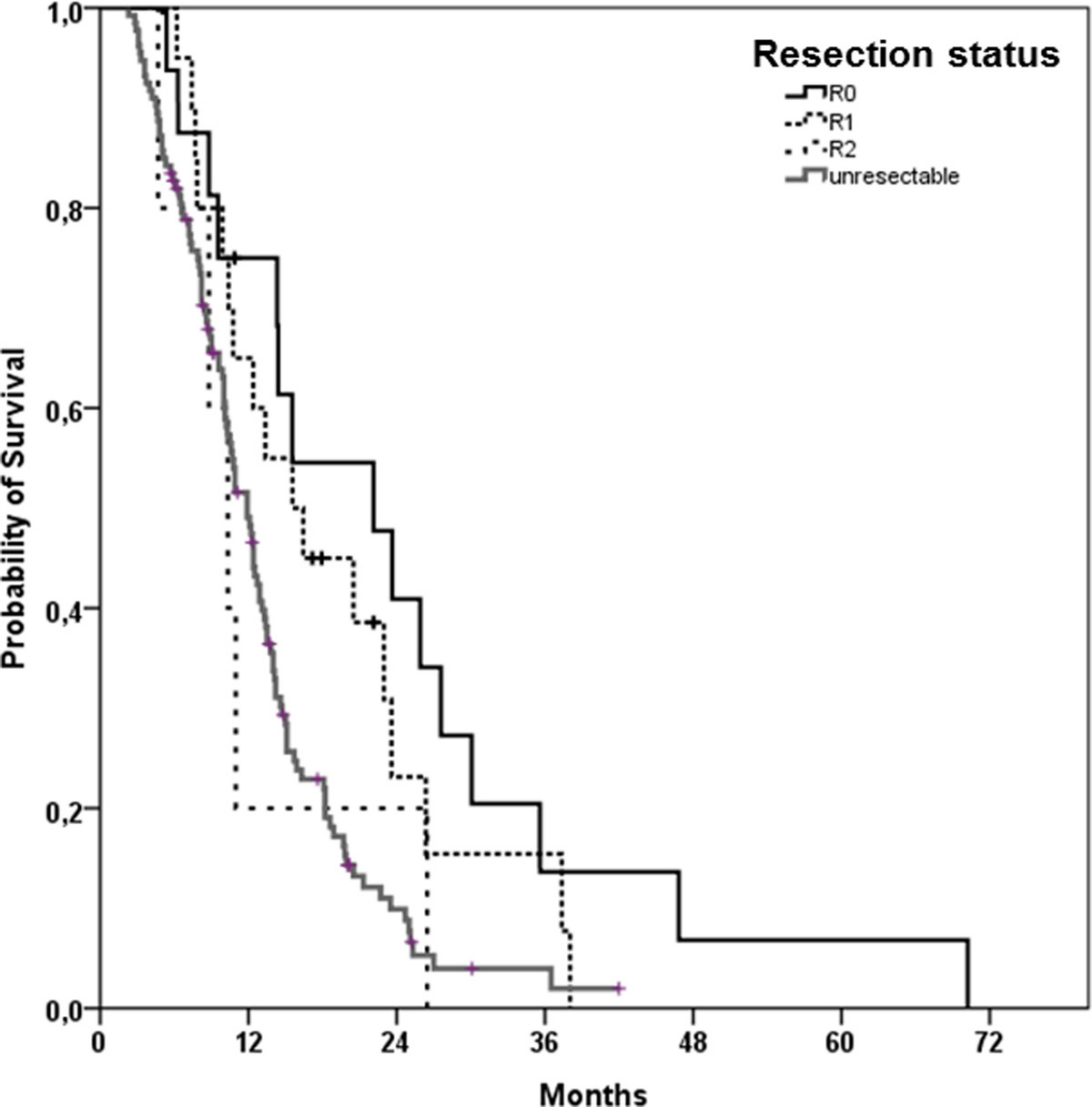
**Kaplan-Meier estimate for overall survival (OS) of patients according to resection status**.

R0-resected patients had a median OS of 22.1 months (95%-CI: 10.7-33.5 months, SD = 5.8), 15.6 months (95%-CI: 8.9-22.3 months, SD = 3.4) in case of R1-resection and 10.3 months (95%-CI: 7.0-13.6 months, SD = 1.7) in R2-resected patients (Figure 4). In the four cases of pathological not definable resection margins (Rx), median OS was comparatively worse with 4.3 months (95%-CI: 2.8-5.9 months, SD 0.79). OS differed significantly between R0-resected patients and unresected patients (p = 0.003) and between R1-resected patients and those not amenable to secondary resection (p = 0.029).

### Patterns of disease progression and disease-free survival

During follow-up patients underwent CT or MRI scans regularly and progression of disease was detected in 105 patients, of which local tumour progression was seen in 36 (34%) and distant metastasis in 89 patients (68%). Both local and systemic progression was seen in 20 patients (19%). Main site of systemic disease progression was the liver (52%) and the peritoneum (36%). As described above a total of 23 metastases were discovered during explorative laparotomy.

**Figure 5:**
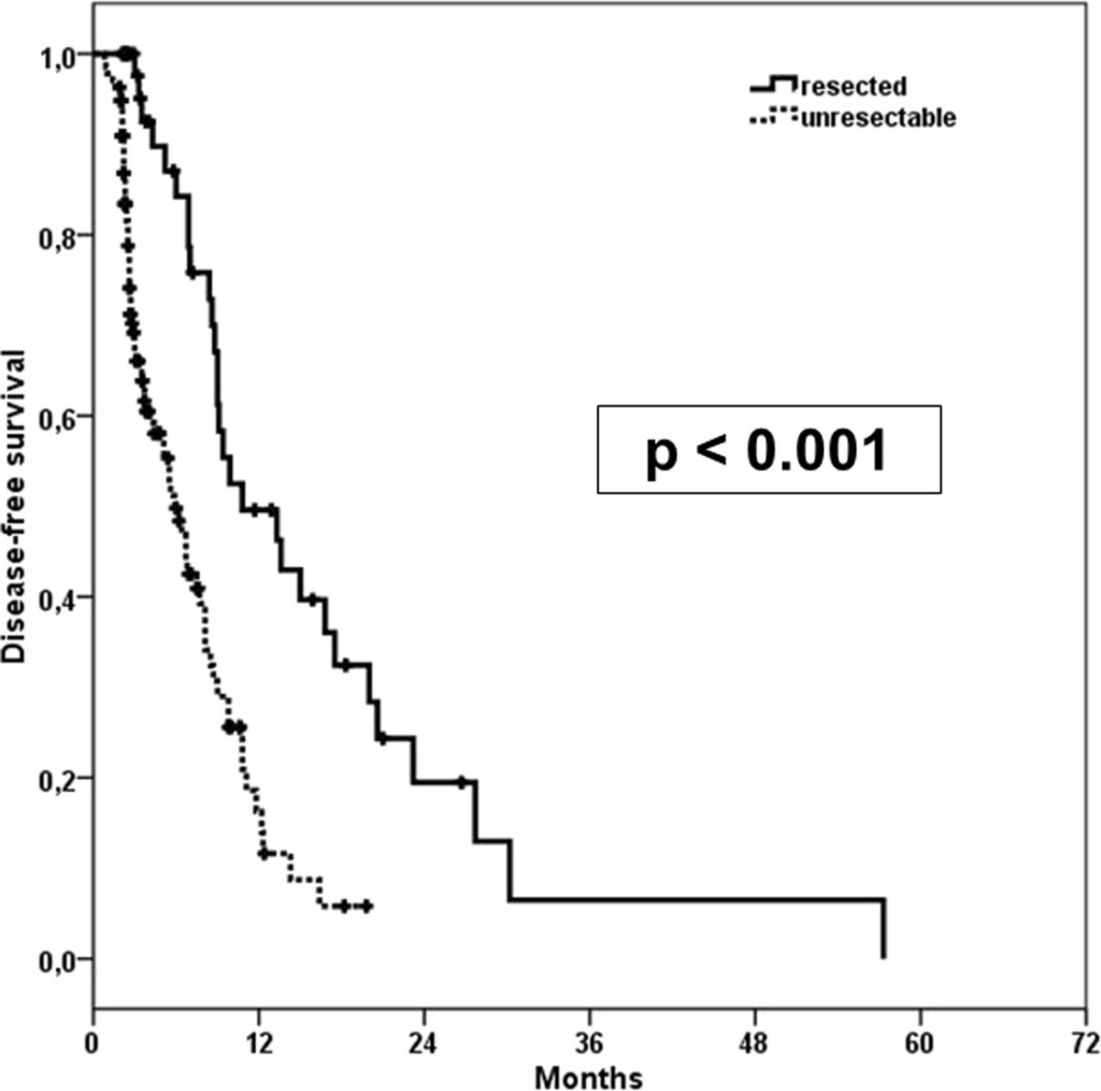
**Kaplan-Meier estimate for disease-free survival (DFS) of resected and unresected patients**.

Median time to disease progression (DFS) was 8.1 months (95%-CI: 6.6-9.6 months, SD = 0.7). There was a significant difference between DFS in resected and unresected patients with 10.8 and 5.9 months respectively (p < 0.001) (Figure 5).

### Toxicity of chemoradiation

Radiation side effects observed were mild in all patients and included nausea, fatigue and diarrhoea. Haematological toxicity attributed to GEM chemotherapy was primarily CTCAE (version 4.03) grade I-III and consisted of thrombocytopenia or neutropenia. As previously described, in one patient duodenal bleeding appeared after CRT due to tumour infiltration or as a consequence of therapy. The patient underwent a radical duodenopancreatectomy with tumour-free resection margins. No further grade IV or V toxicities were observed. Notably in 63% of all patients administration of GEM had to be interrupted for more than one week mainly because of haematological toxicity I°-III°. Cholangitis was recorded in 6 patients during CRT and was clinically manageable with antibiotic and supportive treatment. Skin reactions after GEM application were seen in 3 patients; in one of these patients systemic therapy was discontinued. Gemcitabine-induced pneumonitis was diagnosed in one patient. One patient died 13 days after the end of radiotherapy and thus before the planned restaging.

## Discussion

Neoadjuvant chemoradiation can lead to secondary resectability in patients with LAPC, and patients amenable to resection profit substantially with respect to overall survival. In our group of 215 patients, secondary resection was possible in a high percentage of all patients, and overall survival was increased significantly in these operated patients from a median of 11.9 months to a maximum of 22.1 months in completely resected patients. This fact underlines the high importance of a margin-free resection in pancreatic cancer patients to improve survival.

In the literature, it is reported that about 10-20% of all patients can be treated with a surgical resection directly after diagnosis [10,23]. However, depending on the experience of the surgical center, these numbers may vary substantially. Higher patient load and specialization on pancreatic cancer surgery are associated with higher resectability rates and lower perioperative mortality [24,25], and thus more patients are primarily classified as resectable. This means, on the contrary, that patients with locally advanced disease referred to neoadjuvant chemoradiation for downsizing may represent a subgroup of patients in such a center associated with very large tumours or other negative prognostic factors.

Combined radiation and chemotherapy (CRT) is certainly the most self-evident strategy for tumour downsizing, and the efficacy of this approach has been confirmed for many other tumours in gastrointestinal oncology [3-5]. However, for pancreatic cancer, clinical data have failed so far to indicate which patients may benefit from a neoadjuvant approach. In recent analyses there is growing evidence that supports this therapeutic approach to obtain a higher rate of secondary resectability and prolonged survival [18,19,26].

Gillen et al. have recently summarized 111 studies including 4394 patients that underwent preoperative/neoadjuvant treatments in case of unresectable, resectable and borderline resectable pancreatic cancer [18]. The authors demonstrated that in patients initially resectable, neoadjuvant treatment does not impact resectability, resection status or outcome compared to initial resection and adjuvant treatment. However, in the group of locally advanced and unresectable patients, about 1/3 of the tumours become resectable during the course of treatment. OS was increased from 9.5 months (range 6-12 months) in non-resected patients to 20.5 months (range 9-62 months) in patients undergoing secondary resection.

According to Gillen and colleagues, resectability criteria varied substantially within the included studies [18]: A minority of studies focussed on the NCCN guidelines for resectability [27]. The majority of studies did not report any detailed information on their resectability criteria. This information clearly underlines that decision for resection and classification of patients as locally inoperable depends on the treating center and their expertise and experience in the field. Moreover, this underlines that clinical volume is directly linked to outcome as well as side effects in pancreatic cancer treatment [28-35].

In our analysis, median OS after secondary resection with tumour-free resection margins was 22.1 months, which is significantly higher than OS in the group of non-resected patients (11.9 months); however, compared to similar studies reporting outcome after neoadjuvant treatment [19], survival rates are somewhat lower. Concerning this fact it has to be considered that only patients with unresectable tumours were included in this study. Due to the high throughput of pancreatic cancer patients at our center, it is most likely that patients referred to neoadjuvant treatment are the most difficult candidates for curative resection, associated with negative prognostic factors.

Despite this fact, our data clearly show that over one fourth of the patients are qualified for secondary resection, and those patients significantly benefit with respect to OS and DFS. Our results are in accordance with published data on resection rates after neoadjuvant CRT which are reported to lie between 10-20% (recently reviewed by [36]).

According to current literature one of the main sites of R1 resection in pancreatic head cancer is the mesopancreatic region [37]. Median survival after R1-resection was 15.6 and 5.6 months in R2- and Rx-resected patients (p < 0.05). Importance of resection status was also observed in DFS of different patient groups. While median DFS for all patients was 8.1 months, resected patients had a significant later onset of disease progression with 10.8 months.

Nevertheless, distant metastasis is still observed in a high percentage of pancreatic cancer patients. A recent mono-institutional analysis reports that a majority of 145 curatively resected patients developed distant metastases during follow-up predominantly in the liver or peritoneum rather than a local relapse [38].

Also in our analysis systemic disease progression was more frequent than local progression. Main sites of distant metastasis were the liver (52%) and the peritoneum (36%).

A diagnostic biopsy was not achievable in all of the analyzed patients but nevertheless treatment as described above was initiated according to local guidelines. In these cases, characteristic CT or MRI morphological findings and elevated CA 19-9 levels in the blood serum were required for treatment initiation.

Pancreatic cancer is still having a dismal prognosis and only a minority of patients can undergo curative resection at time of diagnosis. Neoadjuvant GEM-based CRT for LAPC without evidence of distant metastases can lead to a high rate of second resectability resulting in long-term survival and disease-free survival. Nevertheless disease progression is seen in a vast majority of patients, especially occurrence of distant metastases still remains the predominant site of disease progression.

In conclusion, this work confirms the value of neoadjuvant treatment in locally advanced pancreatic cancer observed in a very large group of homogeneously treated patients. Future clinical trials will prove if a neoadjuvant systemic therapy (with gemcitabine) followed by concurrent CRT and subsequent resection will help to identify patients that benefit from an intensified therapy.

## Competing interests

The authors declare that they have no competing interests.

## Authors' contributions

TW, HH, SR, JUW, JEW, MWB, FB, PS DH, JD and SEC were responsible for patient treatment and care. DH collected the patients' data, performed all statistical analyses and wrote the manuscript. KK, TW, HH, AA, FB, PS, SR, JUW, JEW, MWB, PS, MWB, JD and SEC contributed to the analysis of data and revised the manuscript. SEC conceived the study, helped to write and finalized the manuscript. All authors helped with the interpretation of the data, read and approved the final manuscript.
